# Effectiveness and cost-effectiveness of primary arthrodesis versus open reduction and internal fixation in patients with Lisfranc fracture instability (The BFF Study) study protocol for a multicenter randomized controlled trial

**DOI:** 10.1186/s12893-021-01320-1

**Published:** 2021-08-12

**Authors:** N. A. C. van den Boom, G. A. N. L. Stollenwerck, S. M. A. A. Evers, M. Poeze

**Affiliations:** 1 Dept. of Trauma Surgery, Maastricht University Medical Centre, P. Debyelaan 25, 6229 HX Maastricht, The Netherlands; 2 Nutrim School for Nutrition, Toxicology and Metabolism, Maastricht University, Universiteitssingel 40, 6229 ER Maastricht, The Netherlands; 3 Dept. of Surgery-Trauma Surgery, Alrijne Hospital, Simon Smitweg 1, 2353 GA Leiderdorp, The Netherlands; 4 Trimbos Institute, Netherlands Institute of Mental Health and Addiction, Da Costakade 45, 3521 VS Utrecht, The Netherlands

**Keywords:** Traumatic Lisfranc fracture dislocation, ORIF, PA, Quality of life, Cost effectiveness

## Abstract

**Background:**

The Lisfranc injury is a complex injury of the midfoot. It can result in persistent pain and functional impairment if treated inappropriately. In Lisfranc fracture dislocation, treatment options are primary arthrodesis of the midfoot joints or open reduction and internal fixation. The purpose of the proposed study is to define the optimal treatment for the Lisfranc fracture dislocation, either primary arthrodesis or open reduction and internal fixation, in regard to quality of life, complications, functional outcomes, and cost effectiveness.

**Methods:**

Study design: A prospective multicenter RCT. Study population: All patients of 18 years and older with an acute (< 6 weeks) traumatic fracture dislocation in the Lisfranc midfoot joints, displaced on static radiographic evaluation or unstable with dynamic evaluation, weight bearing radiographs or fluoroscopic stress testing under anesthesia, and eligible for either one of the surgical procedures. In total, this study will include n = 112 patients with Lisfranc fracture dislocation. Interventions: Patients with Lisfranc fracture dislocation will be randomly allocated to treatment in “The Better to Fix or Fuse Study” (The BFF Study) with either PA or ORIF. Main study parameters/endpoints: Primary outcome parameter: the quality of life. Secondary outcomes: complications, functional outcomes, secondary surgical interventions and cost effectiveness. Nature and extent of the burden: PA is expected to have a better outcome, however both treatments are accepted for this injury with a similar low risk of complications. Follow up is standardized and therefore this study will not add extra burden to the patient.

**Discussion:**

This study protocol provides a comprehensive overview of the aims and methods of the attached clinical study. Limitations of this study are the absence of patient blinding since it is impossible in surgical intervention, and the outcome measure (AOFAS) that has limited validity not for these injuries. This study will be the first with enough power to define optimal treatment for Lisfranc fracture dislocations. This is necessary since current literature is unclear on this topic.

*Trial registration* Current controlled Trial: NCT04519242 with registration date: 08/13/2020. Retrospectively registered; Protocol date and version: Version 4 05/06/2020

**Supplementary Information:**

The online version contains supplementary material available at 10.1186/s12893-021-01320-1.

## Background

*‘The human foot is a masterpiece of engineering and a work of art’*—Leonardo da Vinci. The foot does indeed consist of an intricate structure of bones and joints, where stability and mobility are balanced during gait. Injuries to the bones or joints of the foot, for example in fracture dislocation of the midfoot joint complex, therefore, have a significant impact on both the stability and mobility of the foot. The midfoot or Lisfranc joints are of great importance for the stability of the foot [1–3]. Injuries at the Lisfranc complex are not common, approximately 0.2% of all fractures with an incidence of 1/55,000 [1, 2]. The impact of these injuries on the function of the foot and the quality of life is, however, considerable. In addition, there are significant healthcare- and societal costs. Finally, these injuries are also indicative of the impact of other injuries of the foot, such as Chopart or intertarsal joint injuries.

“The Better to Fix or Fuse Study” (The BFF Study) is the first prospective large randomized controlled trial (RCT), worldwide comparing the two generally accepted treatment strategies in displaced or unstable Lisfranc fractures: Open Reduction and Internal Fixation (ORIF) and Primary Arthrodesis (PA). Until now there have only been three previous reported RCTs comparing ORIF vs. PA for Lisfranc injuries, and one non-RCT, and eight retrospective case series [4–15].

Overall, these studies concluded that PA seems to result in less secondary surgery, less implant removal, and a faster return to activity. Besides, there is some evidence of a better functional outcome after arthrodesis as reported in the Patient Reported Outcome Measures (PROMs) reported in several of the above-mentioned studies. these results are, however, still not significant [4–15]. Nevertheless, the current overall evidence slightly favors arthrodesis as a primary treatment of dislocated Lisfranc injuries. However, all these results do not reach significant improvement and the risk of bias of these previous studies is either moderate or high.

The BFF Study is also the first study to not only look at the Lisfranc injury when unstable on static radiographic evaluation, but also at the injury with displacement solely at dynamic evaluation. Weight bearing radiographs a/o fluoroscopic stress testing under anesthesia, are included. At the time of publication of this study protocol there is no clear evidence what treatment is best for the non-displaced unstable Lisfranc injury as proven by displacement at dynamic stress testing (Additional file 1).

## Diagnosis and treatment

An inadequately treated Lisfranc injury with persistent instability can result in multiple late complications, most commonly painful instability of the joint malformation or post-traumatic arthritis [16, 17]. All these complications can lead to gait dysfunction and foot pain [16, 17]. Therefore, adequate surgical treatment is needed to restore stability. Due to the diversity of injuries, there is no gold standard for treating all Lisfranc injuries in a similar manner [10]. It is therefore important to distinguish between ligamentous Lisfranc injuries and Lisfranc fracture dislocation.

PA is generally accepted as the treatment of choice in pure ligamentous injuries. In case of an unstable Lisfranc injury, with displacement either on static or dynamic radiographic evaluation, the best treatment is aimed at a stable construction with anatomic alignment [10]. This can be achieved by using either ORIF or by PA. ORIF is associated with problems such as the occurrence of secondary arthritis in 40–90% and the need for secondary interventions to perform arthrodesis or to remove the osteosynthesis materials in order to relieve pain [18, 19]. To prevent this need for secondary interventions and the development of post-traumatic arthritis, primary arthrodesis is suggested [6, 7, 12, 20]. On the other hand, retaining the joints of the midfoot may possibly improve the functional mobility and thereby the quality of life after removal of the osteosynthesis materials. As these potential complications can have a major impact on patients’ everyday lives, research is needed to define the best available surgical treatment for these patients with unstable Lisfranc fracture injury.

## Economic aspects

The need to deliver healthcare efficiently has increased substantially during the last years [21]. Two studies reviewed the costs of ORIF and PA, one of them found PA was significantly more expensive and, in contrast, one found PA to be more cost-effective [21, 22]. These studies measuring the cost-effectiveness only measured the patient costs such as professional care and diagnostic tests. We suggest measuring the social and family costs caused by reduced productivity and hospital visiting as well, since Lisfranc injuries may often cause long-term complaints that may influence these costs [22, 23].

## Evaluation of treatment

A number of studies have already been published comparing ORIF with PA in Lisfranc injuries. Most of these studies have used Patient Reported Outcome Measures (PROMs) and X-ray evaluations to evaluate both treatments. The most common PROM used in Lisfranc studies is the American Orthopaedic Foot and Ankle Score (AOFAS) [10, 21, 24–26]. Other commonly used PROMs include the Short-form 36 or Short-form 12 (SF-36 or SF-12 score) and the Visual Analogue Scale (VAS). As the efficiency of health care interventions become more and more important recent years, the patient health-care costs, the family costs and societal costs have to be investigated too for both treatment options. These costs have not been investigated in previous randomized studies. Our study is, to our knowledge, the first prospective RCT that will investigate these costs for both treatment options.

## Need for this study

Because of the limitations of the methodological quality of the previous reported clinical trials it is still impossible to favour one intervention; PA or ORIF, over the other for the treatment of unstable and or dislocated Lisfranc fractures. Therefore, for drawing a definitive conclusion for the best treatment there is an urgent need for a large prospective high-quality RCT. Such a trial should also assess cost-effectiveness, as cost considerations might be decisive in decision making especially when both treatments are equal based on PROMs.

## Methods

### Aim of the study

The primary objective of this study is to compare the effects ORIF vs. PA have on the quality of life of patients with unstable or displaced Lisfranc fractures over a period of 24 months.

The secondary objectives are to compare the differences in the amount and type of secondary procedures, including removal of osteosynthesis materials, difference in objective and subjective functional outcomes, difference in alignment of the foot on weight-bearing X-rays, difference in occurrence of complications and differences in costs and cost-effectiveness.

### Study design

The BFF Study is a nationally prospective multicenter, randomized controlled trial (RCT) performed in the Netherlands. In total, 13 medical institutions in the Netherlands will enroll patients during a 24 months period, and the enrolment is stared at June 2020. The participating institutions of “The BFF Study” are defined in the Additional file 2.

### Study setting

The BFF Study is a multicenter prospective RCT and will be conducted in The Netherlands in thirteen participating medical institutions. Please see the Additional file 2 for more information about the location of the participating medical institutions. The SPIRIT (Standard Protocol Items: Recommended for Interventional Trials) 2013 statement will be followed. Approval of the local medical ethics committee was obtained with Registration number: NL 73038.096.20. Furthermore, “The BFF Study”-The Better to Fix or Fuse Study, is retrospectively registered in the ClinicalTrials.gov database with Registration number: NCT04519242 on 08/13/2020 and will be conducted in line with the declaration of Helsinki.

### Trial recruitment and allocation

Every medicinal institution participating in “The BFF Study” will have a Local Investigator (LI). This LI is in most cases the treating physician of the patient. The Coordinating Investigator (CI) coordinates the trial and gives advice to all separated LI’s of each medical institution. Together the LI’s of each medical institution, which are all trauma surgeons, and the CI will form “The BFF Study” group. The LI’s will actively screen patients for eligibility at the emergency department or at the outpatient clinic of their institution. If there are any questions about the eligibility criteria the CI will be contacted by the LI. All the LI’s, and side investigators in each medical institution are mentioned in Additional file 2. The CI is also described in Additional file 2.

### Characteristics of participants

Patients can be included if they meet all the following inclusion criteria:

- Age ≥ 18 years.
- Acute Lisfranc fracture injury (< 6 weeks after trauma).
- Displaced or unstable injury with weight-bearing radiographs or fluoroscopic stress testing under anesthesia*.
- Independent for activities of daily living (yes/no question).

Partients who meet one or more of the following criteria will not be considered for inclusion:

- Age < 18 years.
- Open Lisfranc injury.
- Pure ligamentous Lisfranc injury.
- Non-displaced and stable injury with weight-bearing radiographs or stress testing under anaesthesia.
- Contra-indications for general or locoregional anaesthetic techniques.
- Other fractures in the ipsilateral leg.
- Pre-existent abnormalities at the Lisfranc complex.
- Pre-existent immobility.
- Dependent in activities of daily living (due to dementia, Alzheimer’s, NYHA class IV angina and heart failure, oxygen-dependent COPD).
- Rheumatoid arthritis.
- Pathologic fractures (metastasis, secondary osteoporosis).
- Peripheral neuropathy and/or diabetes.
- Alcohol or drug abuse preventing adequate follow-up.

### Definition of displaced or unstable*

Instability, e.g., displacement or malalignment on radiographs interpreted as follows:

- AP view:
  Lateral displacement of 2nd metatarsal on intermediate cuneiform, medial margin of the second metatarsal and the middle cuneiform not aligned. TMT 1 disruption, lateral margin of the first metatarsal not aligned with the lateral margin of the medial cuneiform; Gap between 1st and 2nd metatarsal and/or ceuneiforme medialis and intermedius (> 2 mm);
- 30-degree oblique view:
  Lateral displacement of 3rd metatarsal on lateral cuneiform, lateral margin of the third metatarsal and the lateral cuneiform not aligned; Medial margin of the fourth metatarsal and cuboid not aligned;
- Lateral view:
  Dorsal displacement of the metatarsal bases above the level of the cuneiforms.
- Any view:
  Bony avulsion fractures between base 2nd metatarsal and cuneiforme medialis (fleck sign), and/or between cuneiforme medialis and intermedius.

### Recruitment procedure

**Fig. 1:**
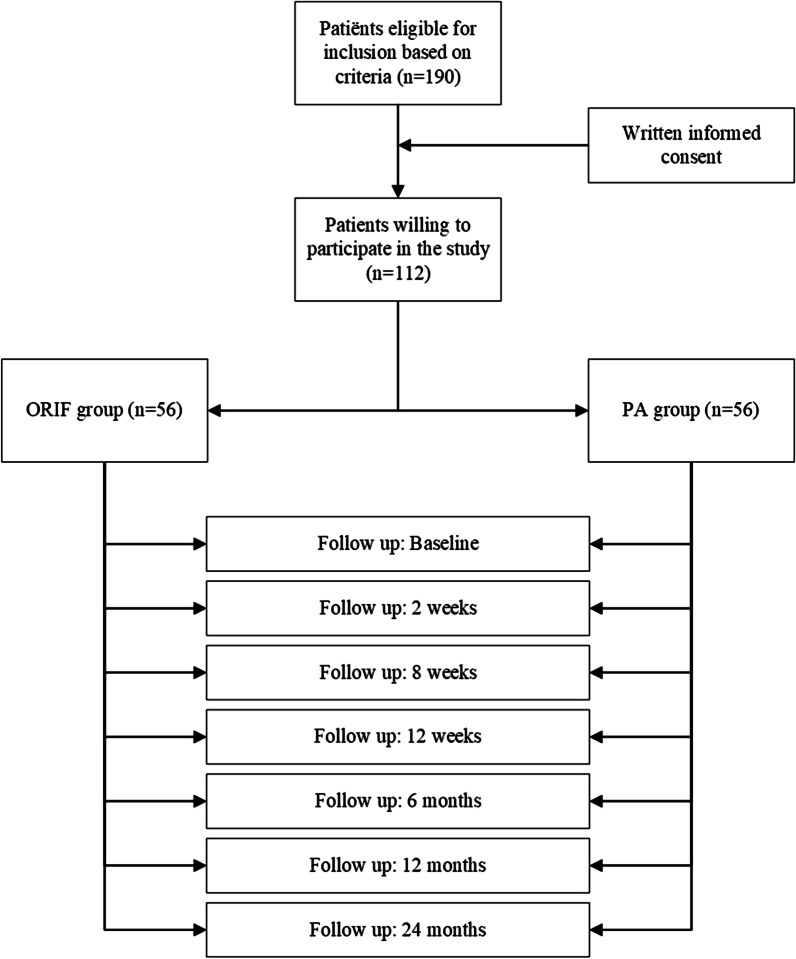
Flow chart describing the recruitment and follow-up process

Patients eligible for inclusion will be recruited by the LI at the emergency department or at the outpatient clinic of each participating medical institution. The LI in each hospital will be contacted about every Lisfranc injury as part of normal clinical practice. Local protocol in the medical institutions will daily revise patient files and X-rays so to decrease the chance that patients with Lisfranc injuries will be missed. The LI will inform the patient about the two surgical treatment options for displaced or unstable Lisfranc fractures. If a patient is eligible for participation in the BFF Study, the LI will provide the potential patient with the Patient Information Folder (PIF) and the Informed Consent (IC) form (Additional file 1). The patient can take the PIF home to read the information carefully. The patient will then have the option to make contact by telephone or mail with the LI or CI to ask questions about the study participation. Patients will be granted a reasonable amount of decision time, at least 7 days, to make a decision if they want to participate (Fig. 1).

Patients will be asked for formal written IC prior to participation in accordance with Good Clinical Practice. Patients who decide to participate will give their written informed consent to the participating medical institution. After permission to participate the patients will be randomly assigned to one of the two treatment arms which is ORIF or PA. The patient will be informed by the LI or CI which type of surgery they will get so they can ask specific questions about this type of surgery.

### Data collection and processing

Baseline characteristics will be received through the electronic patient file (EPF) and clinical assessment by the LI and stored in the case report form (CRF) as set down by the online data management system Castor Electronic Data Capture, an application through which one can easily and safely collect data online.

The EPF is screened every follow-up to assess late complications, re-interventions, re-admissions, duration of medical institution stay and consultations at the medical institution. This screening and follow up assessment will also be done by the LI, or side investigator of the medical institution or by the CI if necessary. All of this data information will also be reported in the CRF by the LI, side investigator or CI. If the LI or side investigator have any questions regarding the data, the CI will be contacted.

### Follow-up procedure

Follow-up will consist of clinical follow-up at 2 weeks and clinical follow-up and e-mail questionnaires at baseline at 8 and 12 weeks, and 6, 12 and 24 months after surgery (Fig. 1). After care will be the same for all patients, namely plaster cast immobilization or Air Walker for 8 weeks in total. Plaster cast immobilization or Air Walker will be non-weight bearing for 4 weeks followed by weight bearing for another 4 weeks.

All questionnaires will be digital. Enrolled patients will receive a link by e-mail at the preset follow-up moments to fill in before the standard follow-up moment at the outpatient clinic occurs, except at 2 weeks post-surgery. These messages are regulated by Castor Electronic Data Capture.

Radiographs will be performed each follow up period and interpreted as “aligned or not aligned” using previously mentioned alignment criteria for that specific follow-up moment.

To promote participants retention and complete follow-up the patients are called by the CI if the participant does not fill in the questionnaire within 5 days after invitation.

### Participant withdrawal

The LI or CI can decide to withdraw a participant from the study for urgent medical reasons. Participants can leave the study at any time for any reason if they wish to do so, without any consequences. Withdrawal of participants will be recorded, including the reason (if known) in the data management system; Castor Electronic Data Capture. The withdraw will also be recorded in the EPF.

### Randomization and blinding

After given written informed consent, participants will be randomized in a 1:1 ratio to PA or ORIF, using randomization stratified by the center with random permuted block sizes of four patients. This randomization will be performed using a web-based computer (L-DOT) directed randomization. This randomization tool was developed by the centre of data and information management at the Faculty of Health, Medicine and Life Sciences of Maastricht University and MUMC+ (MEMIC). A unique study record number will be generated, and the allocation will be disclosed. Due to the comparison of outpatient-based and surgical treatment strategies in this study, blinding to the treatment allocation for participants and medical staff is not possible. A specialized independent statistician will analyze the data blinded for treatment allocation.

## Trial interventions

### Open reduction and internal fixation

ORIF, stabilization without fusion, is one of the surgical interventions in the treatment of Lisfranc injury. After surgical exposure any dislocation will be reduced and fixation of the Lisfranc injury will be achieved by osteosynthesis. Osteosynthesis of the first three TMT joints or intercuneiform joints will preferably be done by using bridge plate (variable angle locking plate and/or locking plate and/or dynamic compression plate—3.5 mm, 2.7 mm or 2.4 mm) and/or trans articular screw osteosynthesis (4.0 mm cannulated screws and/or solid small fragment screws and/or HCS). For fixation TMT4 and/or TMT5 K-wires (1.6–2.0 mm) will be used instead.

### Primary arthrodesis

PA, stabilization by fusion, is the other surgical intervention of choice in the treatment of Lisfranc injury. After surgical exposure, cartilage of the involved unstable joints ray 1–3 of the Lisfranc injury will be removed and after reduction, fixation will be done by osteosynthesis with bone graft if necessary. Osteosynthesis will be done by using bridge plate (variable angle locking plate and/or locking plate and/or dynamic compression plate—3.5 mm, 2.7 mm or 2.4 mm) and/or trans articular screw osteosynthesis (4.0 mm cannulated screws and/or solid small fragment screws and/or HCS).

The tarsometatarsal joints 4–5 are essential joints to remain dynamic and the consensus is to fix these using some temporary fixation device, mostly K-wires if remaining dislocated after arthrodesis of the first 3 TMT joints.

### Potential harms

The sponsor will suspend the study if there are adequate motives to suppose that continuation of this trial will harm a participant’s safety or well-being. This is in accordance with section, subsection 4 of the Medical Research Involving Human Subjects Act (WMO). Subsequently the sponsor will inform the licensed Medical Ethical Board without unreasonable delay of a temporary halt including the reason for such an action. The study will be suspended while waiting for a further positive submission by the Medical Ethical Board. All the included participants of the study kept informed by the CI. The adverse events (AEs) of the study are defined as any unwanted finding occurring to an included participant during the study, whether related to the study or not. This AEs are reported spontaneously by the included participants or observed by the LI or side investigator of each medical institution. All of the serious adverse events will be announced in compliance with the guidance from The Central Committee on Research Involving Human Subjects (CCMO).

## Outcomes

### Primary outcome

The primary outcome is the quality of life as defined by the EQ-5D-5L questionnaire. The EQ-5D-5L questionnaire is used to assess the health-related quality of life in this study. This questionnaire consists of a descriptive method including five health related measurements and a Visual Analogue Scale (VAS) that documented the participants self-determined healthiness. Due to an algorithmic program that is based on the values obtained from the Dutch population the index scores for each included participant of the study can be measured. Subsequently, to calculate the QALY these index scores will be combined with the length [27].

### Secondary outcomes

Secondary outcomes are the differences in the amount and type of secondary procedures (including removal of osteosynthesis materials). The most important complications are post-traumatic arthritis, infection, persistent instability, non-union and foot deformity (pes planovalgus). The frequency of all complications will be measured using post-surgery follow-up visits. The differences in objective and subjective functional outcomes; active range of motion as determined by the online questionnaires: American Orthopaedic Foot and Ankle Society Score (AOFAS), Short Form Health Survey (SF-12), and Foot and Ankle Disability Index (FADI). The range of motion will also be measured at the clinical follow-up visits. The difference in alignment of the foot on weight-bearing X-rays (stability) which will be measured at the clinical follow-up moments. Secondary economic outcomes are the patient, family and societal health care costs, and the cost-effectiveness as measured with the online Medical Consumption Questionnaire (MCQ) and Productivity Costs Questionnaire (PCQ).

## Statistical analysis

### Descriptive statistics

Baseline patient characteristics will be described stratified by group allocation. Continuous variables will be summarized using mean and standard deviation (SD) or as median and first and third quartile. Categorical variables will be summarized as count and percentage.

Missing data will be imputed using stochastic regression imputation, using predictive mean matching to draft the values to be imputed, as this is more robust to misspecification of the imputation model and more appropriate for non-normally distributed continuous variables.

All analyses of the primary and secondary study parameters will be analysed in according with the intention to treat principle. A p-value of ≤ 0.05 will be considered to indicate statistical significance. All analyses will be performed using IBM SPSS version 25 or later.

### Sample size and feasibility

The primary outcome is quality of life over the follow up period, quantified using the EQ-5D-5L score. The longitudinal analysis will include multiple observations per patient, but it is unclear how strong consecutive measurements are correlated. For that reason, we performed a sample size calculation for a cross-sectional contrast between the two groups, so that even with near-perfect correlation, we would have sufficient power to detect a clinically meaningful difference.

The estimated standard deviation (SD) of the EQ-5D-5L scores is 0.15. To be able to have sufficient statistical power (i.e., 80%) to detect a clinically meaningful difference in QoL of 0.08 points, we need to include 56 patients per group, or 112 in total. We estimate, based on previous experience, that we would need to screen approximately 190 patients to find 112 patients eligible and willing to participate.

### Primary study parameter

EQ-5D-5L scores after treatment will be compared between groups using a linear mixed-effects regression model to account for clustering of multiple observations within each patient. Both absolute differences between groups, such as the difference in slope over time will be estimated. All parameters will be reported including 95% confidence interval. Additionally, we will add potential confounders as covariates to the model, irrespective of qualitatively determined baseline imbalance. We will add body mass index (BMI), age, and sex.

### Secondary study parameters

Binary secondary outcome variables (i.e., having received any secondary surgical procedure, experienced any type of complication, showing stability and alignment on weight bearing radiographs) will be compared between groups using Pearson’s Chi-squared test. In case of expected cell counts of less than five we will use Fisher’s Exact test.

Continuous secondary outcomes (i.e., scores on the AOFAS, SF-12, FADI, MCQ, and PCQ) will be analysed using a linear mixed-effects regression model, similar to the analysis of the primary study parameter.

## Economic evaluation

A trial-based economical evaluation will be completed from a societal and healthcare point of view with a total follow-up period of 24 months, and this evaluation is in line with the Dutch guidelines for health economical evaluation [28]. The Dutch costing manual is the source for the valuation of the cost prices [27]. The so called ‘friction cost method’ will be calculating the amount of absence of work of each included participant in this study. This ‘friction cost method’ is recommended by the Dutch manual for costing [29]. The cost-effectiveness in this study will be measured in societal cost per QALY, as based on the EQ-5D-5L questionnaire. Subsequently the sensitivity and the bootstrap analysis will be conducted to notice possible uncertainty. To visualize the chance of the cost-effectiveness of the two interventions cost-effectiveness acceptability curves will be constructed. Subsequently, sensitivity analyses will be performed on the data input for testing the strength of the study results. Besides, in accordance with the Dutch guidelines for economic evaluations as well as the ISPOR guidelines, a budget-impact analysis (BIA) will be performed [30].

## Data

### Data management

The study participants will all receive a special participant identification codification. This codification consists of four capital letters (A, B, etc.) indicating the medical institution of inclusion and is followed by three numbers (001, 002, etc.) indicating the order of inclusion in the study. The BFF study team, the Health Care Inspectorate, and the monitor from the external clinical trial organization; Clinical Trial Management Maastricht (CTCM) will have access to this private participant data. Data will be archived in a password protected digital database. The CI will secure the key to the participant identification codification and the private participant related data. The data will be saved for 15 years after finalization of the study. A full Data Management Plan can be obtained by contacting the CI of the BFF Study.

### Data protection

All data concerning participants or their participation in this trial will be considered confidential and handled in compliance with all appropriate regulations. Only the LI, side investigators, CI and monitor of the study have access to these patient data.

### Data safety monitoring

The CTCM is independent from the sponsor of the study and will be monitoring this study. The duties of the CTCM include checking the IC of each included participant, reported AE’s and SAE’s, participant characteristics, and check the completeness of the electronic CRF and if this CRF is corresponding with the EPF. The monitoring includes one site initiation visit (SIV), one monitoring visit after five participant inclusions in the medical institution and one close out visit per participating medical institution of the study.

## Benefits and risks assessment, group relatedness

Literature indicates that both treatment options in this study, ORIF or PA, are accepted for Lisfranc injury. No clear advantage for one treatment option is found at present in the literature. Both treatment options have their known complications, related to the injury itself and the surgical procedure: stiffness of the joint and pain due to arthritis, persistent instability, nonunion, infection, anesthesiologic complications, and hardware failure. But this risk of specific complications is low and generally similar in both treatment options.

During the normal follow-up visits, patients will have to fill in health questionnaires before the appointment at the hospital and will be seen by the treating physician, the researcher, or an independent research assistant. This will take an extra 5–15 min of the patient’s time per visit to the outpatient clinic. Other aspects of the follow-up are identical to that of standard treatment.

In the Netherlands, the annual incidence of Lisfranc fractures is estimated 0.2% of the total number of 300,000 fractures in the Netherlands [16, 28, 29]. Lisfranc injuries are thereby uncommon, but the impact of this injury to the well-being of the patient is significant [24] and up to 20–30% of injuries can be missed during the initial assessment [30].

If this study shows that performing an PA reduces complications, improves outcome, and reduces costs, standard practice can be changed so as to spare patients the disadvantages, such as reinterventions, longer hospital stays, and/or reduction of societal impact. Direct and indirect healthcare costs will be reduced, resulting in savings of €10.4 million euro per year as calculated [4, 6, 18, 19], as well as further savings if the findings are applied to other fractures of the foot, such as Chopart, intertarsal and subtalar injuries. The findings will also be used to design the first national guidelines for the treatment of foot injuries, leading to enhanced quality and continuity of healthcare.

This study will not only provide data concerning the optimal treatment of fracture dislocations of the midfoot, but this study will also provide a framework for individualized patient centered surgery balancing the advantages and disadvantages of retaining or removing a joint in the foot after trauma. Finally, this study will increase the evidence-based level of guidelines in trauma surgery.

## Compensation for injury

There is no need for a liability insurance for the subjects participating in this study since there is no higher risk when included in the study in comparison with the standard care for this injury. There is only need for a regular liability insurance of the hospitals participating in this study.

## Auditing of the participating medical institutions

Auditing will be performed by the CTCM and will be independent from investigators and the sponsor.

## Protocol amendment

In the case of any modifications of the study protocol that may impact the conduct of the study will be communicated to the Medical Research and Ethics Committee Zuyderland.

## Publication policy

Publication will be in accordance with international recognized scientific and ethical standards concerning publications and authorship, including the Uniform Requirements for Manuscripts Submitted to Biomedical Journals, established by the International Committee of Medical Journal Editors.

## Discussion

This protocol publication presents a prospective, randomized, multicenter trial including 13 participating hospitals in the Netherlands. It gives detailed information of study aim, the study method such as the patient flow, patient randomization procedure and recruitment, treatment aftercare and methods of analysis of the collected data and ways to present and publish the results.

This study contains several limitations. Patient blinding was not possible, depending on the surgical treatment and follow-up. Another limitation is within the outcome measure (AOFAS) which has limited validity for this type of injury. Although this might constitute a limitation, the AOFAS Midfoot Scale is the most commonly used outcome measure for patients treated for Lisfranc injury, providing a tool for comparing other studies. Besides this, the FADI questionnaire is used as additional secondary outcome measurement.

The strength of this study is that this is the first study with enough power (n = 112) to define the optimal treatment for patients specifically with Lisfranc fracture dislocation, either PA or ORIF. The study applies to quality of life, type and number of complications, secondary surgical interventions, functional outcomes, and cost effectiveness with an adequate number of patients.

The current literature generally provides poor methodological quality studies with a limited number of patients, which makes it difficult to favour one intervention over the other. Furthermore, the current literature does not investigate specific Lisfranc fracture injuries. To our knowledge there are no previous reported studies or running studies at present which explicitly investigate the unstable Lisfranc fracture injury. In the BFF Study not only the displaced injuries at static radiographic evaluation, but also the non-displaced injuries with instability at dynamic radiographic evaluation, weight bearing radiographs or fluoroscopic stress testing under anesthesia, are also included. At last, the cost measurement, including family and societal costs is not included in previous studies. Cost considerations might be decisive in decision making especially when both treatments are equal based on PROMs. This study will have enough power to determine the optimal treatment for the unstable Lisfranc fracture injury, displaced at static a/o dynamic radiographic evaluation, with the best cost-effectiveness.

## Supplementary Information

**Additional file 1.** Participant information The BFF Study, Information about the study for participants.

**Additional file 2.** Appendix for BMC Surgery. Participating Medical Centers. Local Investigator and Side Investigator names of the included Medical Centers.

## Data Availability

Not applicable.

## Abbreviations

AE: Adverse events
AOFAS: American Orthopedic Foot and Ankle Society Score
BIA: Budget impact analysis
BMI: Body Mass Index
CI: Central Investigator
CRF: Case report form
CTCM: Clinical Trial Centre Maastricht
EPF: Electronic patient file
FADI: Foot and Ankle Dislocation Index
IC: Informed consent
LI: Local Investigator
MCQ: Medical Consumption Questionnaire
ORIF: Open reduction and internal fixation
PA: Primary arthrodesis
PCQ: Patient Consumption Questionnaire
PIF: Patient Information Folder
PROM: Patient Reported Outcome Measure
QALY: Quality Adjusted Life Year
SAE: Serious adverse events
SD: Standard deviation
TMT: Tarsal–MetaTarsal
VAS: Visual Analogue Scale
