# Supplementary material for: Effectiveness and cost-effectiveness of primary arthrodesis versus open reduction and internal fixation in patients with Lisfranc fracture instability (The BFF Study) study protocol for a multicenter randomized controlled trial
Source: BMC Surg. 2021 Aug 12;21:323. doi: 10.1186/s12893-021-01320-1 (PMC8359057; doi:10.1186/s12893-021-01320-1)
Supplement: Supplementary file 1 — Additional file 1. Participant information The BFF Study, Information about the study for participants. [file 12893_2021_1320_MOESM1_ESM.docx]

Subject information for participants in scientific medical research.

**The ‘BFF-study – Better to Fix or Fuse Study, Lisfranc study**

*Retaining or removing the joint in fracture dislocations of the foot? A randomized controlled trial of primary arthrodesis versus joint stabilization in Lisfranc injuries.*

**Inleiding**

Dear Sir/ Madam,

We kindly ask you to participate in a medical scientific study.

Participation is voluntary. Your written permission is required to participate. Before making the decision to participate, it is important to know more about the study. You are receiving this letter because you have suffered a Lisfranc injury to the foot: a fracture of one or more of your tarsus bones and / or metatarsal bones around the Lisfranc joint (see image 1). You will be operated on for this. This letter will explain what the research entails. Please read this information carefully and ask the researcher for an explanation if you have any questions. You can also ask the independent expert named at the end of this letter for additional information. You can also talk about it with your partner, friends or family[
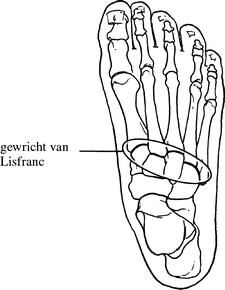
](NULL)

Figure 1: Lisfranc joint

[
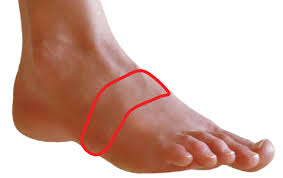
](NULL)

heeft dit onderzoek goedgekeurd.

1. **General information**

This study was set up by the Maastricht University Medical Center + (MUMC +), and is carried out by surgeons / doctors in various hospitals in the Netherlands. A total of nine hospitals in the Netherlands will participate in this study. For this study we want to examine 112 patients with Lisfranc lesions.

(The medical ethics review committee METC Z has approved this study)

1. **Purpose of the study**

The purpose of this study is to determine the most effective surgery for the Lisfranc injury. There are currently two different types of surgery for this injury, namely the fusion of the affected joints in the foot with removal of the cartilage (arthrodesis) and the temporary fixation of the affected joints in the foot by means of fixation material while preserving the cartilage. (joint stabilization). The aim of the study is to investigate which operating technique allows the patient to recover best with as few complications as possible, such as pain complaints. The most cost-effective procedure will also be taken into account.

1. **Background of the study**

According to the current guideline there is no 'gold standard' for the treatment of Lisfranc fractures. Both surgical techniques are accepted treatments where there is no evidence to date that either of the two is preferable. Often the operator decides which type of surgery to choose based on his personal experience. Thus, to date, there is no scientifically proven benefit to either of the two types of surgery.

If you decide to participate in this study, you will be assigned to either the arthrodesis group (allowing the joint to grow together) or the joint stabilization group (temporary fixation of the joints involved) by drawing lots. Both you and the operator know before the operation for which group you have been drawn. This gives you the time to request additional information about this from your practitioner / operator.

1. **What participation means**

Participation in the study takes approximately 24 months. A few days before the operation and five times after the operation (after 8 weeks, 12 weeks, 6 months, 12 months and 24 months) you will be asked to complete an online questionnaire and come to the hospital for a check-up, where you will also receive a check-up X-ray of the foot.

If you do not participate in this study, you will also receive most of these check-up appointments and X-rays; you now come to the hospital no more than one or two extra times.

**Exam:**

You are eligible to participate in the study if the following criteria apply to your situation:

 You are 18 years or older

 Your injury is not older than 6 weeks

 You were fully mobile before this injury

 You do not have rheumatism or known osteoarthritis of the foot

 You do not have a pathological fracture (i.e. on the basis of disease of the affected bone)

 You are independent in your daily life in terms of activities

 The attending practitioner has assessed the X-rays and has concluded that the lesion has been displaced in such a way that it is recommended that it be operated on. *

* When in doubt about the degree of displacement of the fracture, a loaded X-ray of the foot is taken. This is a picture taken while standing on the affected foot, possibly compared to the healthy foot. This investigation is therefore only carried out in case of doubt about the degree of displacement. Sometimes this examination is only done during the operation. Only then will we know whether you can participate in this study.

**Different from usual care**

The visits that are part of this examination do not replace the regular visits / follow-up to your doctor, but there is extra attention and control with regard to your recovery process during this examination.

**5. What is expected of you**

In order for the research to run smoothly it is important that you adhere to the following agreements.

**The agreements are that you:**

• keep appointments for visits with your doctor;

• completes the online questionnaires completely at the appropriate times.

**It is important that you contact the researcher:**

• if you are admitted or treated in a hospital;

• if you suddenly develop health problems;

• if you no longer wish to participate in the study;

• if your contact details change.

**Visits and measurements:**

• Before the operation:

- completing an online questionnaire (± 20 minutes)

• 2 weeks after the operation:

- wound check in hospital; it will be checked whether the wound has healed properly and any non-dissolvable stitches will be removed from the operation wound

• 8 weeks after the operation:

- completing an online questionnaire (± 20 minutes)

- coming to the hospital for your follow-up appointment, including an X-ray to check your foot (± 30 minutes)

- the cast will be removed from the foot and you can start to put weight on the foot without any aids due to the pain

• 3 months after the operation:

- completing an online questionnaire (± 20 minutes)

- coming to the hospital for your follow-up appointment, including an X-ray to check your foot (± 30 minutes)

• 6 months after surgery:

- completing an online questionnaire (± 20 minutes)

- come to the hospital for your check-up appointment, including an X-ray to check your foot (± 30 minutes)

• 12 months after the operation:

- fill out an online questionnaire (± 20 minutes)

- come to the hospital for your check-up appointment, including an X-ray to check your foot (± 30 minutes)

• 24 months after surgery:

- completing an online questionnaire (± 20 minutes)

- coming to the hospital for your check-up, including an X-ray to check your foot ( ± 30 minutes)

It is important that you complete the online questionnaires prior to surgery or your follow-up appointment with your treating physician. You will receive a link for this by e-mail in due course.

If you do not participate in this study, you will also receive most of these check-up appointments and X-rays; you now come to the hospital no more than one or two extra times.

**6. What complications can you experience?**

To date there is no scientific evidence that there is a difference in the complications that can occur with the two types of surgery: arthrodesis (allowing the joint to fuse) versus joint stabilization (temporary fixation of the involved joints). Both operations have complications related to the surgical procedure and the fracture itself, such as:

- Stiffness of the joints in the foot

- Pain due to osteoarthritis of the joints in the foot

- (Wound) infection

- Complications of the medication used for the operation

- Failure of the devices (screws, plates and / or wires) that are used during the operation to fasten the bone parts to each other

- Complaints about the surgical material used (screws, plates and / or wires)

The risk of the above specific complications is very low and is comparable between the two operations.

**Radiation exposure**

During this research we use X-rays. The total radiation exposure for this study does not deviate from the usual (regular) radiation exposure if you do not participate in this study.

**7. Possible pros and cons**

It is important that you consider the possible pros and cons before deciding to participate.

To date there is no scientific evidence that one operation has more or fewer advantages or disadvantages than another operation. If you participate in this study, it does not necessarily mean that you will directly benefit from this. But you do contribute to more knowledge about the treatment of Lisfranc injuries. In addition, you will experience extra attention and extensive follow-up during your recovery period. Participation in the study also means:

• (Extra) tests; online questionnaires;

• That you have agreements that you must adhere to;

By participating in this study, you will have to come for a check-up once or twice more often than is normally the case. In addition, one or two extra X-rays of your feet will have to be taken in order to be able to make a statement about healing, standing and osteoarthritis in the long term. The amount of radiation from the one or two extra X-rays of your feet is so low that no harmful effects are to be expected.

**8. If you do not want to participate or want to stop the study**

You decide whether you want to participate in the study. Participation is voluntary.

If you do not wish to participate, you will be treated for your injury in the usual way using the technique preferred by your operator. Your practitioner will tell you more about the treatment options available and their advantages and disadvantages.

Even if you do not want to participate in the entire study, you can still complete the online questionnaires for this study to evaluate your recovery process.

You can give your consent for this by signing the consent form at the end of this information letter (Appendix C).

These online questionnaires will then have to be completed 4 times in total:

• Before surgery

• After 6 months

• After 12 months

• After 24 months

If you decide to participate in the full study or the online questionnaire study, you can always change your mind and stop anyway, even during the study. You will then be treated again in the usual way for your injury. You don't have to say why you are quitting. You must, however, report this immediately to the researcher. The data collected up to that point will be used for the research.

If new information about the study becomes available that is important to you, the investigator will let you know. You can then decide for yourself whether you want to continue to participate in the study.

**9. End of the study**

Your participation in the study stops if:

 all visits are over;

 you choose to stop.

**10. Use and storage of your data**

For this research your personal data will be collected, used and stored. This concerns information such as your name, address, date of birth and information about your health. The collection, use and storage of your data is necessary to answer the questions asked in this study and to publish the results. We ask your permission for the use of your data.

**Confidentiality of your data**

To protect your privacy, your data is given a code. Your name and other data that can directly identify you are omitted. Data can only be traced back to you with the key to the code. The key to the code remains safely stored in the local research facility. The data sent to the client only contains the code, but not your name or other data with which you can be identified. The data cannot be traced back to you in reports and publications about the research either.

**Access your data for verification**

Some individuals at the research site may have access to all of your data. Also to the data without code. This is necessary to be able to check whether the research has been carried out properly and reliably. Persons who have access to your data for inspection are: the committee that monitors the safety of the research, and a controller / monitor who works for the researcher or who is hired by the researcher. They keep your information secret. We ask you to give permission for this access.

**Retention period of data**

Your data must be kept at the research location for 15 years.

**Withdrawing permission**

You can always withdraw your permission for the use of your personal data. The research data collected up to the moment you withdraw your consent will still be used in the research.

**More information about your rights when processing data**

For general information about your rights when processing your personal data, you can consult the website of the Dutch Data Protection Authority.

If you have any questions about your rights, please contact the person responsible for processing your personal data. See Appendix A for these contact details.

If you have any questions or complaints about the processing of your personal data, we recommend that you first contact the research location. You can also contact the institution's Data Protection Officer. See Appendix A for these contact details.

**11. Insurance for test subjects**

If you participate in the study you run the same risks as with the usual treatment for your condition. The researcher is therefore not required to take out additional insurance from the METC (Medical Ethical Review Committee).

**12. No reimbursement for participation**

The extra tests and treatment for the examination will cost you nothing. You will not be paid for participating in this study.

**13. Do you have any questions?**

If you have any questions, please contact your attending physician or the principal investigators: Drs Stollenwerck and Drs. Van den Boom from “The BFF Study”. For independent advice about participation in this study, please contact the national independent doctor: Dr. Melenhorst (surgeon at MUMC +). He knows a lot about the research, but is not involved in the research as a researcher.

If you have any complaints about the examination, you can discuss this with your attending physician. If you prefer not to do this, you can turn to the complaints committee of your hospital. All information for this can be found in Appendix A.

**14. Signing of the consent form (Appendix B or C)**

When you have had sufficient reflection time (7 days), you will be asked to decide whether to participate in this study. If you consent, we will ask you to confirm it in writing on the accompanying consent form (Appendix B or C). By your written consent, you indicate that you have understood the information and agree to participate in the study.

Both you and the researcher will receive a signed version of this consent form.

Thank you for your attention.

**Appendix A: contact details**

First contact person:

Local principal investigator:

Position:

Email address:

Main Researcher: Drs.Stollenwerck

Position: Principal Investigator "The Bff Study"

Email address: gstollenwerck@alrijne.nl

Main Researcher: Drs. Van den Boom

Position: Coordinating researcher

Email address: n.vandenboom@maastrichtuniversity.nl

Independent physician: Dr. J. Melenhorst

Position: Surgeon, MUMC +

Email address: j.melenhorst@mumc.nl

**Appendix B:**

**Consent form participant full study The BFF Study**

The 'BFF study - Better to Fix or Fuse study', Lisfranc study:

• I have read the information letter . I could also ask questions. My questions have been sufficiently answered. I had enough time to decide whether to participate.

• I know that participation is voluntary. I also know that I can decide at any time not to participate or to stop the study. I don't have to give a reason for that.

• I know that some people may be able to access all of my data for the purposes of audit. Those people are listed in this information letter. I consent to such access by these persons.

• □ I do give

□ I do not give consent to keep my personal data for 15 years and use it for future research on my condition.

• □ I do give

□ I do not give consent to the collection and use of my data to answer the research question in this study.

• I want to participate in this study.

**Participant name:**

Signature: Date: __ / __ / __

-------------------------------------- -------------------------------------------------- -------------------------

I declare that I have fully informed this participant about the said study.

If information becomes known during the study that could influence the consent of the participant, I will inform him / her in good time.

**Name researcher (or his representative):**

Signature: Date: __ / __ / __

--------------------------------- -------------------------------------------------- ------------------------------

**Additional information is provided by:**

Name:

Position:

Signature: Date: __ / __ / __

**Appendix C: Consent Form Participant (online) questionnaires**

**If the patient wants to meet the above investigation not to participate, or not meeting discussed the inclusion criteria of the above research option with patient following:**

• I want to / can not participate in the study called : The 'BFF study - Better to Fix or Fuse study', Lisfranc study, but have no objection to completing the online questionnaires at measurement time 0 (OK date), after 6 months, after 12 months and after 24 months and

• □ I do give

□ I do not give consent to keep my personal data for 15 years and use it for future research on my condition.

• □ I do give

□ I do not give consent to the collection and use of my data to answer the research question in this study.

**Participant name:**

Signature: Date: __ / __ / __

-------------------------------------- -------------------------------------------------- -------------------------

I declare that I have fully informed this participant about the said study.

If information becomes known during the study that could influence the consent of the participant, I will inform him / her in good time.

**Name researcher (or his representative):**

Signature: Date: __ / __ / __

--------------------------------- -------------------------------------------------- ------------------------------

**Additional information is provided by:**

Name:

Position:

Signature: Date: __ / __ / __

**Appendix B:**

**Consent form participant full study The BFF Study**

The 'BFF study - Better to Fix or Fuse study', Lisfranc study:

• I have read the information letter . I could also ask questions. My questions have been sufficiently answered. I had enough time to decide whether to participate.

• I know that participation is voluntary. I also know that I can decide at any time not to participate or to stop the study. I don't have to give a reason for that.

• I know that some people may be able to access all of my data for the purposes of audit. Those people are listed in this information letter. I consent to such access by these persons.

• □ I do give

□ I do not give consent to keep my personal data for 15 years and use it for future research on my condition.

• □ I do give

□ I do not give consent to the collection and use of my data to answer the research question in this study.

• I want to participate in this study.

**Participant name:**

Signature: Date: __ / __ / __

-------------------------------------- -------------------------------------------------- -------------------------

I declare that I have fully informed this participant about the said study.

If information becomes known during the study that could influence the consent of the participant, I will inform him / her in good time.

**Name researcher (or his representative):**

Signature: Date: __ / __ / __

--------------------------------- -------------------------------------------------- ------------------------------

**Additional information is provided by:**

Name:

Position:

Signature: Date: __ / __ / __

**Appendix C: Consent Form Participant (online) questionnaires**

**If the patient wants to meet the above investigation not to participate, or not meeting discussed the inclusion criteria of the above research option with patient following:**

• I want to / can not participate in the study called : The 'BFF study - Better to Fix or Fuse study', Lisfranc study, but have no objection to completing the online questionnaires at measurement time 0 (OK date), after 6 months, after 12 months and after 24 months and

• □ I do give

□ I do not give consent to keep my personal data for 15 years and use it for future research on my condition.

• □ I do give

□ I do not give consent to the collection and use of my data to answer the research question in this study.

**Participant name:**

Signature: Date: __ / __ / __

-------------------------------------- -------------------------------------------------- -------------------------

I declare that I have fully informed this participant about the said study.

If information becomes known during the study that could influence the consent of the participant, I will inform him / her in good time.

**Name researcher (or his representative):**

Signature: Date: __ / __ / __

--------------------------------- -------------------------------------------------- ------------------------------

**Additional information is provided by:**

Name:

Position:

Signature: Date: __ / __ / __
