# Supplementary material for: Effectiveness and cost-effectiveness of primary arthrodesis versus open reduction and internal fixation in patients with Lisfranc fracture instability (The BFF Study) study protocol for a multicenter randomized controlled trial
Source: BMC Surg. 2021 Aug 12;21:323. doi: 10.1186/s12893-021-01320-1 (PMC8359057; doi:10.1186/s12893-021-01320-1)
Supplement: Supplementary file 2 — Additional file 2. Appendix for BMC Surgery. Participating Medical Centers. Local Investigator and Side Investigator names of the included Medical Centers. [file 12893_2021_1320_MOESM2_ESM.docx]

**Coordinating Investigator (CI) : Drs. Van den Boom**

E-mail: [noortje.vanden.boom@mumc.nl](mailto:noortje.vanden.boom@mumc.nl)

**Maastricht University Medical Center (MUMC+)**

Local Investigator (LI): Prof.dr.Poeze

E-mail: [m.poeze@mumc.nl](mailto:m.poeze@mumc.nl)

**Academisch Medical Center (AMC)**

LI: Dr. Schepers

E-mail: t.schepers@amsterdamumc.nl

Side Investigator (SI) Drs. J.A. Halm

E-mail: [j.a.halm@amsterdamumc.nl](mailto:j.a.halm@amsterdamumc.nl)

**Amphia Hospital Breda**

LI: Dr. Schormans

E-mail: [pschormans@amphia.nl](mailto:pschormans@amphia.nl)

**Haaglanden Medical Center (HMC)**

LI: Dr. Hoogendoorn

E-mail: j.hoogendoorn@haaglandenmc.nl

**Radboud University Medical Center (RUMC)**

LI: Dr. Hermans

E-mail: erik.hermans@radboudumc.nl

**University Medical Center Groningen (UMCG)**

LI: Dr. M.El Moumni

E-mail: [m.el.moumni@umcg.nl](mailto:m.el.moumni@umcg.nl)

**Viecuri Medical Center Venlo**

LI: Dr. Janzing

E-mail: hjanzing@gmail.com

**Zuyderland Medical Center**

LI Dr. van Vugt

E-mail: [r.vanvugt@zuyderland.nl](mailto:r.vanvugt@zuyderland.nl)

**Alrijne Hospital**

LI: Drs.Stollenwerck

E-mail: [gstollenwerck@alrijne.nl](mailto:gstollenwerck@alrijne.nl)

**Maasstad Hospital**

LI: Dr. van der Vlies

E-mail: [vliesc@maasstadziekenhuis.nl](mailto:vliesc@maasstadziekenhuis.nl)

SI: Drs. Stassen

**Rijnstate Hospital**

LI: Dr. Kolkman

E-mail: kkolkman[@rijnstate.nl](mailto:j@rijnstate.nl)

**Elisabeth TweeStede Hospital**

LI: Drs. Theeuwes

Email: [hp.theeuwes@ETZ.nl](mailto:hp.theeuwes@ETZ.nl)

**Catharina Hospital**

LI: Dr. Van der Veen

E-mail: [alexander.vd.veen@catharinaziekenhuis.nl](mailto:alexander.vd.veen@catharinaziekenhuis.nl)
